# Susceptibility to Invasive Meningococcal Disease: Polymorphism of Complement System Genes and *Neisseria meningitidis* Factor H Binding Protein

**DOI:** 10.1371/journal.pone.0120757

**Published:** 2015-03-23

**Authors:** Declan T. Bradley, Thomas W. Bourke, Derek J. Fairley, Raymond Borrow, Michael D. Shields, Peter F. Zipfel, Anne E. Hughes

**Affiliations:** 1 Centre for Public Health, School of Medicine, Dentistry and Biomedical Sciences, Queen’s University Belfast, Belfast, United Kingdom; 2 Public Health Agency, Belfast, United Kingdom; 3 Centre for Infection and Immunity, School of Medicine, Dentistry and Biomedical Sciences, Queen’s University Belfast, Belfast, United Kingdom; 4 Regional Virus Laboratory, Belfast Health and Social Care Trust, Belfast, United Kingdom; 5 Vaccine Evaluation Unit, Public Health England, Manchester, United Kingdom; 6 Inflammation Sciences Research Group, School of Translational Medicine, University of Manchester, Manchester, United Kingdom; 7 Department of Infection Biology, Leibniz Institute for Natural Product Research and Infection Biology—Hans Knöll Institute, Jena, Germany; 8 Friedrich Schiller University, Jena, Germany; 9 Formerly of Centre for Public Health, School of Medicine, Dentistry and Biomedical Sciences, Queen’s University Belfast, Belfast, United Kingdom; University of Kentucky College of Medicine, UNITED STATES

## Abstract

**Background:**

*Neisseria meningitidis* can cause severe infection in humans. Polymorphism of Complement Factor H (CFH) is associated with altered risk of invasive meningococcal disease (IMD). We aimed to find whether polymorphism of other complement genes altered risk and whether variation of *N*. *meningitidis* factor H binding protein (fHBP) affected the risk association.

**Methods:**

We undertook a case-control study with 309 European cases and 5,200 1958 Birth Cohort and National Blood Service cohort controls. We used additive model logistic regression, accepting *P*<0.05 as significant after correction for multiple testing. The effects of fHBP subfamily on the age at infection and severity of disease was tested using the independent samples median test and Student’s T test. The effect of *CFH* polymorphism on the *N*. *meningitidis* fHBP subfamily was investigated by logistic regression and Chi squared test.

**Results:**

Rs12085435 A in *C8B* was associated with odds ratio (OR) of IMD (0.35 [95% CI 0.19–0.67]; P = 0.03 after correction). A *CFH* haplotype tagged by rs3753396 G was associated with IMD (OR 0.56 [95% CI 0.42–0.76], *P* = 1.6x10^−4^). There was no bacterial load (*CtrA* cycle threshold) difference associated with carriage of this haplotype. Host *CFH* haplotype and meningococcal fHBP subfamily were not associated. Individuals infected with meningococci expressing subfamily A fHBP were younger than those with subfamily B fHBP meningococci (median 1 vs 2 years; *P* = 0.025).

**Discussion:**

The protective *CFH* haplotype alters odds of IMD without affecting bacterial load for affected heterozygotes. *CFH* haplotype did not affect the likelihood of infecting meningococci having either fHBP subfamily. The association between *C8B* rs12085435 and IMD requires independent replication. The *CFH* association is of interest because it is independent of known functional polymorphisms in *CFH*. As fHBP-containing vaccines are now in use, relationships between *CFH* polymorphism and vaccine effectiveness and side-effects may become important.

## Introduction

The complement system is a fundamental part of the innate immune response. This pathway harms unprotected surfaces by a powerful positive feedback cycle that injures cells by perforating them with circular polymers (the membrane attack complex) and by activating further immune response by releasing opsonins and anaphylatoxins [1–3]. Complement activation can cause harm to both unprotected self and foreign cell surfaces [1,2].

Pathogenic bacteria evade the complement system by mimicking or binding to protective host proteins [4]. Human complement factor H (CFH) is the major inhibitory regulator of the complement system. Polymorphism of *CFH* and the adjacent homologous *CFHR1–5* genes is associated with susceptibility to several inflammatory diseases [5–10]. A genome-wide association study of susceptibility to invasive meningococcal disease identified a major risk association at the *CFH* and *CFHR3* locus [11]. The report noted that the associated variants are in strong linkage disequilibrium with the minor allele of rs1065489 (D936E) in the *CFH* gene, but evidence that this is the functional cause is lacking. Unexpectedly, the associated *CFH* polymorphism as one with no known functional effect and is not one associated with other inflammatory diseases.

*Neisseria meningitidis* infection causes sepsis and meningitis, with death in approximately 10% of cases [12]. Factor H-binding protein (fHBP) and Neisserial Surface Protein A bind host CFH to protect *N*. *meningitidis* [13–15]; *Neisserial* fHBP is critical for meningococcal survival in blood [16]. It binds short consensus repeats 6 and 7 of human CFH, which is a region of CFH that also binds to self-surface membranes [17]. It may cause its severe systemic effects by sequestering host CFH, leaving self surfaces unprotected [17,18]. The common CFH Y402H polymorphism (rs1061170), which is a major risk factor for age-related macular degeneration, is adjacent to the fHBP binding site, but does not affect binding to fHBP [17].

Factor H binding protein has been a recent focus of interest because it is now a component of vaccines against serogroup B *N*. *meningitidis* [19,20], one of which is already used in outbreak control [21] and is likely to be added to the UK childhood immunisation schedule [22], which might result in meningococcal disease becoming rare.

Polymorphism of fHBP can be categorized by two different systems of nomenclature. Fletcher *et al*. use a system of two subfamilies, A and B [19]. Masignani *et al*. use a system of three variant groups (1, 2 and 3) [20]. Disease severity is associated with polymorphic variation affecting the five segments that make up the modular structure of fHBP [23–25]. Previous studies of fHBP sequenced the whole gene from cultured *N*. *meningitidis* isolates, and then defined the subfamily, variant group or modular group using only a small number of sequence features. Our study is the first to define fHBP type directly using DNA isolated from patient blood. This may avoid bias due to variation in the success in culturing different strains of *N*. *meningitidis*.

The aim of this study was to explore the relationship between invasive meningococcal disease and variation of the human complement system. We sought to refine the risk association at the *CFH* locus and to investigate whether variation of *N*. *meningitidis* fHBP affects this association. We explored other variations of the complement system, including terminal pathway genes where deficiency of proteins has been associated with susceptibility to recurrent meningococcal disease [26–29] and two complement inhibitors to which *N*. *meningitidis* binds: *CD46*, which encodes membrane cofactor protein, a membrane-bound complement inhibitor [30,31] and *C4 binding protein*, which inhibits the classical pathway [32].

## Methods

### Ethics Statement

The study was approved by the Office for Research Ethics Committees Northern Ireland (part of the UK National Research Ethics Service; study reference: 10/NIR03/24). Following formal proposal to its project advisory group, the Health Protection Agency (now part of Public Health England) provided anonymised residual clinical diagnostic DNA samples from PCR-confirmed cases of invasive meningococcal disease collected in 2009 and 2010. No identifying information was provided. Informed consent was not required for these samples because only anonymised demographic data and residual clinical DNA samples were provided. The Wellcome Trust Case Control Consortium (WTCCC) 1958 Birth Cohort and National Blood Service samples were collected with informed consent and used with permission of the WTCCC.

### Study Population

The case population characteristics have been described in detail previously [33]. The cases were 309 European individuals with PCR-confirmed invasive meningococcal disease. We did not have access to details of the clinical features or demographic details other than age at time of illness. The *N*. *meningitidis* serogroups were: B, 292; C, 3; W, 4; Y 4. The ages ranged between one month and 73 years, with a median of two years. European ancestry was ascertained by using an ancestry-informative panel of polymorphisms [34] and cluster analysis, as described previously [33].

The control population comprised 5,200 individuals with European ancestry from the United Kingdom 1958 Birth Cohort and National Blood Service (NBS) cohort, for whom microarray genome-wide data were provided by the Wellcome Trust Case-Control Consortium (WTCCC). The WTCCC exclusions were applied, and validated by principal components analysis, as described previously [33]. The case population does not overlap with any group tested in the Davila *et al*. genome-wide association study. The control group is identical to that used in the genome-wide association study [11]. The median age of NBS participants was 45 years. The 1958 Birth Cohort participants were aged 52 years at the time of genotyping.

### Detection of *N*. *meningitidis*

Confirmation of invasive meningococcal disease was based on a positive diagnostic Taqman assay for capsular transfer gene (*ctrA*) at the Health Protection Agency (now Public Health England) Meningococcal Reference Unit using an Applied Biosystems 7700 sequence detection system, as described previously [35]. The cycle number at which each positive sample was detected was reported. Cycle threshold, which is inversely correlated with bacterial load, has been used as an indicator of disease severity in other studies [36,37].

### Genotyping of *CFH* Polymorphisms

Six SNPs in *CFH* were genotyped by SNaPshot primer extension methodology, involving PCR to amplify sequence fragments containing polymorphisms of interest, ExoSAP-IT to neutralise unincorporated dNTPs, fluorescent primer extension, further shrimp alkaline phosphatase clean-up, and analysis on an ABI 3100 genetic analyser. The SNPs genotyped were rs1061170 (Y402H), rs800292 (I62V), rs6677604 (which is in full linkage disequilibrium with the deletion of *CFHR3-CFHR1*), rs3753396 (which is in full linkage disequilibrium with rs1065489, D936E), rs419137, and rs2284664. SNPs were chosen on the basis of our established protocols for genotyping [8, 38]. Oligonucleotides are shown in S1 Table. Genotype calls were made using GeneMarker v1.5.1. The control dataset had been genotyped on an Illumina 1.2M duo SNP microarray. Four core *CFH* haplotypes were defined using four SNPs (Table 1) based our previous studies (with the very closely related haplotypes 1 and 2 in our haplotype model [38] replaced by haplotype A in the present report, defined by rs1061170) [8,38].

**Table 1:** *CFH* Haplotype Definitions.

Haplotype Name	rs1061170	rs3753396	rs6677604	rs2284664
**A**	G	A	G	G
**B**	A	G	G	G
**C**	A	A	A	G
**D**	A	A	G	A

### Sequenom iPLEX for complement gene polymorphisms

Polymorphisms of complement pathway genes were investigated using the Sequenom iPLEX platform. PCR and primer extension oligonucleotides were designed using the My Sequenom Online Tools (Sequenom Inc., San Diego, CA, USA), with a target PCR fragment length of between 80 and 120 bp and target primer extension oligonucleotide of between 15 and 30 bases, according to the manufacturer’s protocol. Oligonucleotide sequences are shown in S2 Table. Shrimp alkaline phosphatase was used to neutralise unincorporated dNTPs after the PCR reaction and the iPLEX Gold resin used for final conditioning after primer extension. The reaction product was nanodispensed onto an array chip by technical staff in the Queen’s University Belfast Genome Core using the MassARRAY nanodispenser prior to operation of the MassARRAY mass spectrometer. Genotyping was then carried out using TYPER software (Sequenom). Each SNP cluster plot was inspected individually for quality of clustering and the mass spectrometry plot examined for potentially erroneous base calls, which were recalled or set to missing if a problem was observed. Data were exported from TYPER and converted to PLINK format using Microsoft Excel 2010.

### Genotyping of *Neisserial fHBP*

A method based on the sequence variation described by Pajon *et al*. [23] was developed and validated by sequencing *fHBP* from eight cultured isolates of *N*. *meningitidis*. We used a one-step duplex of two PCR reactions in the same experiment. Subfamily A and B fHBP were distinguished by detection of a ten-base insertion/deletion polymorphism in module A of the gene. One pair of primers, one of which was fluorescently Fam-labelled, was used to make a product of either 157 or 167 bases in length, which was detected on the ABI 3100 Genetic Analyser. Further distinguishing between variant 2 and variant 3 was by the use of three sequence-specific primers, all paired with one common, Fam-labelled primer. The three sequence-specific primers were of different lengths, and bound to the junction between modules C and D, each of which may have one of two variants. According to Pajon *et al*., three variants are found, and therefore, the sequence-specific primers were designed to bind to these combinations of polymorphic modules C and D. Oligonucleotide sequences are shown in S3 Table. In addition to the subfamily and variant classifications of fHBP, the method can determine the most common modular groups described in Pajon *et al*. [23]. The rare (<0.5%) groups VII, VIII and IX cannot each be distinguished from closely related more common modular groups (Table 2).

**Table 2:** Subfamily, Variant and Modular fHBP groups.

Subfamily	Subfamily A				Subfamily B	
Variant	Variant 2		Variant 3		Variant 1	
**Common Modular Groups**	III	VI	II	V	I	IV
**Rare Modular Groups not differentiated from above by fHBP test**			VIII	IX	VII	

### Statistical Analysis

Assessment for deviation from Hardy Weinberg equilibrium was conducted using the method of Wigginton *et al*. [39] as implemented in PLINK v1.07 [40,41]. A *P* value for deviation of <0.05 in cases, controls, or overall was considered to be significant.

A minimum genotyping rate per SNP of 90% and minimum genotyping rate per individual of 90% were used. Association with individual SNPs was assessed by univariate logistic regression (additive model) in PLINK. Significance for association was accepted at *P* < 0.05 after Bonferroni correction for multiple testing in the study of the complement pathway. Correction for multiple testing was not used for analysis of *CFH* as this was a replication of a previous report.

Individuals missing genotypes for any of the four *CFH* haplotype-tagging SNPs were excluded from haplotype analysis. Multivariate logistic regression was conducted in SPSS v19 for case-control status using the number of copies of each *CFH* haplotype as covariates, omitting one haplotype (D) as a reference variable to avoid multicollinearity.

Cycle threshold was compared between *CFH* haplotype B carriers and non-carriers using Student’s T test in R v3.1.1. Age was compared between fHBP Subfamily A and fHBP Subfamily B using the independent samples median test in SPSS.

## Results

### CFH

The genotyping rate was 99.7%. No markers deviated significantly from Hardy Weinberg equilibrium. Two cases failed genotyping entirely, no other individuals were excluded for incomplete genotyping and no markers were excluded because of low genotyping.

Rs1061170 G was associated with increased risk (OR 1.26 [95% CI 1.07–1.49], *P* = 5.3x10^–3^) and rs3753396 G was associated with reduced risk (OR 0.56 [95% CI 0.43–0.74], *P* = 3.0x10^–5^) (Table 3). Multivariate logistic regression shows that haplotype B, tagged by rs3753396 G, is significantly associated with a reduced odds ratio of invasive meningococcal disease (OR 0.56 [95% CI 0.42–0.76], *P* = 1.6x10^–4^) (Table 4). Haplotype C, which carries the deletion of *CFHR3* and *CFHR1* [8] was not associated with the phenotype.

**Table 3:** Association between *CFH* SNPs and IMD.

SNP	Allele 1	Allele 1 Frequency in Cases	Allele 1 Frequency in Controls	Allele 2	Number of cases	Number of controls	Odds Ratio (95% Confidence Interval)	*P*
rs800292	A	0.26	0.23	G	307	5199	1.16 (0.96–1.40)	0.13
rs1061170	G	0.43	0.38	A	306	5124	1.26 (1.07–1.49)	5.3x10^−3^
rs6677604	A	0.19	0.20	G	307	5190	0.95 (0.77–1.17)	0.61
rs3753396	G	0.10	0.17	A	304	5196	0.56 (0.43–0.74)	3.0x10^−5^
rs419137	C	0.15	0.13	A	307	5200	1.22 (0.97–1.53)	0.09
rs2284664	A	0.23	0.21	G	307	5199	1.14 (0.94–1.38)	0.20

**Table 4:** Additive Model Multivariate Logistic Regression for *CFH* haplotype and IMD.

	Odds Ratio	*P*
Haplotype A (rs1061170 [G])	1.06 (0.87–1.29)	0.57
Haplotype B (rs3753396 [G])	0.56 (0.42–0.76)	1.6x10^−4^
Haplotype C (rs6677604 [A])	0.89 (0.70–1.15)	0.37

The cycle threshold for *CtrA* in haplotype B carriers was 31.4 and for ‘wild-type’ non-carriers was 31.3.

### Factor H-Binding Protein Characteristics in the Case Population

The frequencies of the *Neisserial* fHBP variants are shown (Table 5). These are similar to those reported by Pajon *et al*.[23].

**Table 5:** fHBP Variant Frequencies in the Case Population.

fHBP Module Group	Present Study Number (frequency)	Pajon[23] Number (frequency)
I/VII	129 (0.52)	126 (0.52)
II/VIII	7 (0.03)	21 (0.09)
III	12 (0.05)	26 (0.11)
IV	54 (0.22)	12 (0.05)
V/IX	24 (0.10)	32 (0.13)
VI	20 (0.08)	25 (0.10)
Total	246	242
**fHBP Variant Group**		
1	183 (0.74)	138 (0.57)
2	32 (0.13)	51 (0.21)
3	31 (0.13)	53 (0.22)
Total	246	242
**fHBP Subfamily**		
A	71 (0.26)	104 (0.43)
B	198 (0.74)	138 (0.57)
Total	269	242

The median age of cases with subfamily A fHBP *N*. *meningitidis* was one year and that of cases with subfamily B fHBP *N*. *meningitidis* was two years (independent-samples median test *P* = 0.025). The mean cycle threshold for detection of *ctrA* for cases with subfamily A fHBP *N*. *meningitidis* was 29.7 and that for subfamily B fHBP *N*. *meningitidis* was 29.2.

### Susceptibility to *N*. *meningitidis* with Subfamily A and Subfamily B fHBP

*CFH* Haplotype B was associated with a statistically significant protective effect against the rarer subfamily A *fHBP*-expressing *N*. *meningitidis* infection, but was not associated with protection against the more common subfamily B fHBP-expressing *N*. *meningitidis* (Table 6). However, there was no significant difference between the distribution of fHBP subfamily *N*. *meningitidis* infection in haplotype B heterozygote cases and wild-type homozygote cases (Pearson chi-square *P* = 0.11; Table 7).

**Table 6:** Association of *CFH* Haplotypes with Susceptibility to Invasive Infection with *N*. *meningitidis* expressing Subfamily A and Subfamily B fHBP.

	Subfamily A fHBP		Subfamily B fHBP	
	Odds Ratio (95% CI)	P	Odds Ratio (95% CI)	P
Haplotype A [rs1061170 G]	1.31 (0.83–2.05)	0.25	1.03 (0.78–1.36)	0.83
Haplotype B [rs3753396 G]	0.46 (0.22–0.98)	0.046	0.72 (0.50–1.06)	0.09
Haplotype C [rs6677604 A]	0.99 (0.56–1.74)	0.98	0.91 (0.65–1.28)	0.59

**Table 7:** Direct Comparison of Subfamily of Haplotype B Heterozygotes and Non-Heterozygotes (Wild-type).

	Subfamily A	Subfamily B
Haplotype B Heterozygote	9	42
Wild-type Homozygote	49	120

### Complement Pathway

Twenty nine individuals were excluded because of a low genotyping rate, and no SNPs were excluded because of low genotyping leaving 281 cases and 5,199 controls. The remaining genotyping rate was 99.8%. Five SNPs were excluded due to significant deviation from Hardy Weinberg equilibrium in controls, all of which were in *CD46*.

One SNP in each of *CD46* (rs2796278), *C5* (rs17216529), *C8A* (rs17300936) and *C8B* (rs12085435) was associated with the phenotype before correction for multiple testing (Table 8). The association with rs12085435 in *C8B* was significant after Bonferroni correction for 21 tests (*P* = 0.03), and the other SNP associations were non-significant. Both *C8* SNPs were independently associated with the disease phenotype in multivariate logistic regression (Table 9).

**Table 8:** Sequenom iPLEX of Complement Pathway Genes in IMD.

Chromosome	Position(NCBI b37)	SNP Name	Gene	Change	Allele 1	Cases	Controls	Allele 2	Odds Ratio (95% CI)	P
1	57064136	rs947636	*C8A*		C	0.32	0.29	T	1.15 (0.96–1.38)	0.14
1	57113315	rs652785	*C8A*	Q>K	T	0.42	0.38	G	1.19 (1.00–1.41)	0.06
1	57155946	rs17300936	*C8A*	P>L	A	0.09	0.13	G	0.67 (0.50–0.89)	6.2x10^−3^
1	57180072	rs626457	*C8B*		A	0.34	0.33	G	1.04 (0.87–1.25)	0.67
1	57187898	rs12085435	*C8B*	P>L	A	0.02	0.05	G	0.35 (0.19–0.67)	1.3x10^−3^
1	57195072	rs1013579	*C8B*	G>R	G	0.03	0.03	A	0.96 (0.58–1.61)	0.89
1	57195099	rs12067507	*C8B*	E>K	T	0.04	0.05	C	0.71 (0.46–1.10)	0.13
1	205356059	rs2842704	*C4BPA*		C	0.14	0.14	T	1.03 (0.80–1.32)	0.83
1	205360403	rs4425986	*C4BPA*		C	0.44	0.43	T	1.05 (0.88–1.25)	0.58
1	205364303	rs1126618	*C4BPA*	G>G	T	0.17	0.16	C	1.06 (0.84–1.33)	0.61
1	205371523	rs4844573	*C4BPA*	I>T	C	0.32	0.35	T	0.90 (0.75–1.08)	0.26
1	205372211	rs4571969	*C4BPA*		T	0.24	0.24	C	1.00 (0.82–1.23)	0.99
1	206022446	rs2796278	*CD46*		C	0.54	0.49	A	1.25 (1.05–1.48)	0.01
5	39285479	rs155375	*C9*		T	0.43	0.44	C	0.96 (0.81–1.13)	0.61
5	39400311	rs700233	*C9*	R>W	A	0.37	0.40	G	0.88 (0.74–1.05)	0.16
5	40998620	rs10941528	*C7*		A	0.23	0.23	G	1.01 (0.83–1.24)	0.90
5	41001075	rs3805221	*C7*		T	0.23	0.24	C	0.99 (0.81–1.21)	0.89
5	41021361	rs4957361	*C7*		T	0.35	0.36	C	0.98 (0.82–1.17)	0.79
5	41235716	rs1801033	*C6*	A>E	C	0.35	0.37	A	0.94 (0.79–1.13)	0.52
9	122765747	rs17612	*C5*	E>D	C	0.06	0.08	A	0.74 (0.51–1.06)	0.10
9	122840039	rs17216529	*C5*	V>I	A	0.04	0.07	G	0.55 (0.36–0.84)	5.6x10^−3^

**Table 9:** Multivariate logistic regression of rs17300936 and rs12085435.

SNP	OR (95% CI)	P
rs17300936 A	0.65 (0.48–0.88)	5.4x10^−3^
rs12085435 A	0.34 (0.18–0.64)	8.4x10^−4^

## Discussion

Our study explored the characteristics of the association between meningococcal disease and *CFH* polymorphism, the relationship between fHBP subfamily and this association and the effects of other polymorphisms of complement system genes on disease risk.

Our findings provide further evidence supporting the association between *CFH* polymorphism and invasive meningococcal disease reported by Davila *et al*. [11] but as we used the same control group as that study, this is not a full independent replication of the finding. Haplotypic exploration revealed that the association is due to only one haplotype (B), which does not carry any of the known major functional variants associated with AMD. The haplotypes that carry deletion of *CFHR3-CFHR1* (haplotype C) and Y402H (haplotype A) were not associated in a multivariate logistic regression. The protective haplotype B was not associated with any difference in the bacterial load (measured by cycle threshold), suggesting that while disease risk is altered by carrying this variant, severity of disease is not. There was no significant difference in age of cases between carriers and non-carriers of haplotype B, suggesting that the protective mechanism does not affect the age of onset.

There was no significant difference between the distribution of the fHBP subfamilies in individuals who have a copy of *CFH* haplotype B and those who do not, which suggests that *CFH* haplotype variation does not alter the risk of infection with meningococci with fHBP subfamilies A and B differently.

The patients affected by *N*. *meningitidis* with fHBP subfamily A were significantly younger than those who had infection with *N*. *meningitidis* with fHBP subfamily B.

Our investigation of other complement pathway polymorphisms suggests that that a coding polymorphism in *C8B* may be associated with susceptibility to invasive meningococcal disease. This is consistent with the observation that terminal complement component deficiencies increase risk of invasive meningococcal disease [42]. This possibility requires replication in an independent cohort.

Our study is unique in integrating human and bacteriological genomic information to assess the effects of variation of genes that produce interacting proteins. The most important limitation of this study is the lack of an independent replication group to confirm the association at *C8B*. Our study used the same control group as Davila *et al*. [11], meaning that we have not reported a full independent replication of the association at *CFH*. We conducted our own laboratory experiments to genotype meningococcal disease cases and compared them to controls that were genotyped using a different method, at a different time. This presents risk of a systematic genotyping error that could result in a false association. Independent replication is therefore vital. The exploration of the effect of *CFH* haplotype on disease severity (indicated by *CtrA* cycle threshold) and of *fHBP* on severity and age, are not affected by these limitations because these are analyses of cases only.

The functional basis for the relationship between *CFH* polymorphism and susceptibility to invasive meningococcal disease is not yet understood: It is likely that it relates to the interaction between CFH or CFH-related proteins and *N*. *meningitidis*. As meningococcal disease becomes less common, the focus of research may change to questions of immunity and vaccination: It would be most interesting to understand whether vaccine fHBP interacts differently with different variants in host CFH and CFHR proteins following vaccination in humans, and whether vaccination results in complement activation through transient sequestration of CFH. Costa *et al*. recently suggested that fHBP with low affinity for CFH should be explored in future development of fHBP-based vaccines to increase immunogenicity and reduce the chance of autoantibody formation to CFH [43]. It is conceivable that carriage of the protective *CFH* haplotype could influence the effectiveness or side-effects of the vaccine, such as fever, which is common following meningococcal B vaccination [44].

The association between *C8B* rs12085435 and risk of meningococcal disease in our study is in keeping with the effect of inherited terminal complement deficiencies on susceptibility to meningococcal disease [42]. Independent replication will be key to establishing whether *C8B* is a second complement gene associated with meningococcal disease risk.

## Supporting Information

S1 Table *CFH* PCR and SNaPshot Oligonucleotides. (DOCX)

S2 Table Sequenom iPLEX Oligonucleotides. (DOCX)

S3 Table fHBP Oligonucleotides. (DOCX)

## References

[1] Zipfel PF. Complement and immune defense: From innate immunity to human diseases. Immunol Lett. 2009;126: 1–7. 10.1016/j.imlet.2009.07.005 19616581

[2] Zipfel PF, Skerka C. Complement regulators and inhibitory proteins. Nat Rev Immunol. 2009;9: 729–740. 10.1038/nri2620 19730437

[3] Bradley DT, Zipfel PF, Hughes AE. Complement in age-related macular degeneration: A focus on function. Eye. 2011;25: 683–693. 10.1038/eye.2011.37 21394116 PMC3178140

[4] Zipfel PF, Hallstrom T, Hammerschmidt S, Skerka C. The complement fitness factor H: Role in human diseases and for immune escape of pathogens, like pneumococci. Vaccine. 2008;26: 67–74. 10.1016/j.vaccine.2008.11.015 19388168

[5] Klein RJ, Zeiss C, Chew EY, Tsai JY, Sackler RS, Haynes C, et al Complement factor H polymorphism in age-related macular degeneration. Science. 2005;308: 385–389. 15761122 10.1126/science.1109557 PMC1512523

[6] Haines JL, Hauser MA, Schmidt S, Scott WK, Olson LM, Gallins P, et al Complement factor H variant increases the risk of age-related macular degeneration. Science. 2005;308: 419–421. 15761120 10.1126/science.1110359

[7] Hageman GS, Anderson DH, Johnson LV, Hancox LS, Taiber AJ, Hardisty LI, et al A common haplotype in the complement regulatory gene factor H (HF1/CFH) predisposes individuals to age-related macular degeneration. Proc Natl Acad Sci U S A. 2005;102: 7227–7232. 15870199 10.1073/pnas.0501536102 PMC1088171

[8] Hughes AE, Orr N, Esfandiary H, Diaz-Torres M, Goodship T, Chakravarthy U. A common CFH haplotype, with deletion of CFHR1 and CFHR3, is associated with lower risk of age-related macular degeneration. Nat Genet. 2006;38: 1173–1177. 16998489 10.1038/ng1890

[9] Zipfel PF, Edey M, Heinen S, Jozsi M, Richter H, Misselwitz J, et al Deletion of complement factor H-related genes CFHR1 and CFHR3 is associated with atypical hemolytic uremic syndrome. PLoS Genet. 2007;3: e41 17367211 10.1371/journal.pgen.0030041 PMC1828695

[10] Zhao J, Wu H, Khosravi M, Cui H, Qian X, Kelly JA, et al Association of genetic variants in complement factor H and factor H-related genes with systemic lupus erythematosus susceptibility. PLoS Genet. 2011;7: e1002079 10.1371/journal.pgen.1002079 21637784 PMC3102741

[11] Davila S, Wright VJ, Khor CC, Sim KS, Binder A, Breunis WB, et al Genome-wide association study identifies variants in the CFH region associated with host susceptibility to meningococcal disease. Nat Genet. 2010;42: 772–776. 10.1038/ng.640 20694013

[12] Ramsay M, Kaczmarski E, Rush M, Mallard R, Farrington P, White J. Changing patterns of case ascertainment and trends in meningococcal disease in England and Wales. Commun Dis Rep CDR Rev. 1997;7: R49–54. 9127510

[13] Madico G, Welsch JA, Lewis LA, McNaughton A, Perlman DH, Costello CE, et al The meningococcal vaccine candidate GNA1870 binds the complement regulatory protein factor H and enhances serum resistance. J Immunol. 2006;177: 501–510. 16785547 10.4049/jimmunol.177.1.501 PMC2248442

[14] Lewis LA, Ngampasutadol J, Wallace R, Reid JEA, Vogel U, Ram S. The meningococcal vaccine candidate neisserial surface protein A (NspA) binds to factor H and enhances meningococcal resistance to complement. PLoS Pathog. 2010;6: e1001027 10.1371/journal.ppat.1001027 20686663 PMC2912398

[15] Lewis LA, Carter M, Ram S. The relative roles of factor H binding protein, neisserial surface protein A, and lipooligosaccharide sialylation in regulation of the alternative pathway of complement on meningococci. J Immunol. 2012;188: 5063–5072. 10.4049/jimmunol.1103748 22504643 PMC3345070

[16] Dunphy KY, Beernink PT, Brogioni B, Granoff DM. Effect of factor H-binding protein sequence variation on factor H binding and survival of neisseria meningitidis in human blood. Infect Immun. 2011;79: 353–359. 10.1128/IAI.00849-10 21041484 PMC3019890

[17] Schneider MC, Prosser BE, Caesar JJ, Kugelberg E, Li S, Zhang Q, et al Neisseria meningitidis recruits factor H using protein mimicry of host carbohydrates. Nature. 2009;458: 890–893. 10.1038/nature07769 19225461 PMC2670278

[18] Markiewski MM, DeAngelis RA, Lambris JD. Complexity of complement activation in sepsis. J Cell Mol Med. 2008;12: 2245–2254. 10.1111/j.1582-4934.2008.00504.x 18798865 PMC2673539

[19] Fletcher LD, Bernfield L, Barniak V, Farley JE, Howell A, Knauf M, et al Vaccine potential of the neisseria meningitidis 2086 lipoprotein. Infect Immun. 2004;72: 2088 Infection and Immunity-2100. 15039331 10.1128/IAI.72.4.2088-2100.2004 PMC375149

[20] Masignani V, Comanducci M, Giuliani MM, Bambini S, Adu-Bobie J, Aricò B, et al Vaccination against neisseria meningitidis using three variants of the lipoprotein GNA1870. J Exp Med. 2003;197: 789–799. 12642606 10.1084/jem.20021911 PMC2193853

[21] Ladhani SN, Cordery R, Mandal S, Christensen H, Campbell H, Borrow R, et al. Preventing secondary cases of invasive meningococcal capsular group B (MenB) disease: Benefits of offering vaccination in addition to antibiotic chemoprophylaxis to close contacts of cases in the household, educational setting, clusters and the wider community. 2014; Available: https://www.gov.uk/government/publications/invasive-meningococcus-capsular-group-b-menb-preventing-secondary-cases

[22] Joint Committee on Vaccination and Immunisation. JCVI position statement on use of Bexsero meningococcal B vaccine in the UK. 2014; Available: https://www.gov.uk/government/publications/meningococcal-b-vaccine-jcvi-position-statement

[23] Pajon R, Beernink PT, Harrison LH, Granoff DM. Frequency of factor H-binding protein modular groups and susceptibility to cross-reactive bactericidal activity in invasive meningococcal isolates. Vaccine. 2010;28: 2122–2129. 10.1016/j.vaccine.2009.12.027 20044056 PMC2830318

[24] Beernink PT, Granoff DM. The modular architecture of meningococcal factor H-binding protein. Microbiology. 2009;155: 2873–2883. 10.1099/mic.0.029876-0 19574307 PMC2859308

[25] Piet JR, Brouwer MC, Exley R, van der Veen S, van de Beek D, van der Ende A. Meningococcal factor H binding protein fHbpd184 polymorphism influences clinical course of meningococcal meningitis. PLoS One. 2012;7: e47973 10.1371/journal.pone.0047973 23110143 PMC3479137

[26] Brouwer MC, Read RC, van de Beek D. Host genetics and outcome in meningococcal disease: A systematic review and meta-analysis. Lancet Infect Dis. 2010;10: 262–274. 10.1016/S1473-3099(10)70045-1 20334849

[27] Brouwer MC, de Gans J, Heckenberg SG, Zwinderman AH, van der Poll T, van de Beek D. Host genetic susceptibility to pneumococcal and meningococcal disease: A systematic review and meta-analysis. Lancet Infect Dis. 2009;9: 31–44. 10.1016/S1473-3099(08)70261-5 19036641

[28] Dale AP, Read RC. Genetic susceptibility to meningococcal infection. Expert Rev Anti Infect Ther. 2013;11: 187–199. 10.1586/eri.12.161 23409824

[29] Ram S, Lewis LA, Rice PA. Infections of people with complement deficiencies and patients who have undergone splenectomy. Clin Microbiol Rev. 2010;23: 740–780. 10.1128/CMR.00048-09 20930072 PMC2952982

[30] Johansson L, Rytkonen A, Bergman P, Albiger B, Kallstrom H, Hokfelt T, et al CD46 in meningococcal disease. Science. 2003;301: 373–375. 12869763 10.1126/science.1086476

[31] Källström H, Liszewski MK, Atkinson JP, Jonsson A. Membrane cofactor protein (MCP or CD46) is a cellular pilus receptor for pathogenic neisseria. Mol Microbiol. 1997;25: 639–647. 9379894 10.1046/j.1365-2958.1997.4841857.x

[32] Jarva H, Ram S, Vogel U, Blom AM, Meri S. Binding of the complement inhibitor C4bp to serogroup B neisseria meningitidis. J Immunol. 2005;174: 6299–6307. 15879129 10.4049/jimmunol.174.10.6299

[33] Bradley DT, Bourke TW, Fairley DJ, Borrow R, Shields MD, Young IS, et al Genetic susceptibility to invasive meningococcal disease: MBL2 structural polymorphisms revisited in a large case-control study and a systematic review. Int J Immunogenet. 2012;39: 328–337. 10.1111/j.1744-313X.2012.01095.x 22296677

[34] Phillips C, Salas A, Sanchez JJ, Fondevila M, Gomez-Tato A, Alvarez-Dios J, et al Inferring ancestral origin using a single multiplex assay of ancestry-informative marker SNPs. Forensic Sci Int Genet. 2007;1: 273–280. 10.1016/j.fsigen.2007.06.008 19083773

[35] Guiver M, Borrow R, Marsh J, Gray SJ, Kaczmarski EB, Howells D, et al Evaluation of the applied biosystems automated taqman polymerase chain reaction system for the detection of meningococcal DNA. FEMS Immunol Med Microbiol. 2000;28: 173–179. 10799809 10.1111/j.1574-695X.2000.tb01473.x

[36] Darton T, Guiver M, Naylor S, Jack DL, Kaczmarski EB, Borrow R, et al Severity of meningococcal disease associated with genomic bacterial load. Clin Infect Dis. 2009;48: 587–594. 10.1086/596707 19191644

[37] Hackett SJ, Guiver M, Marsh J, Sills JA, Thomson APJ, Kaczmarski EB, et al Meningococcal bacterial DNA load at presentation correlates with disease severity. Arch Dis Child. 2002;86: 44–46. 11806883 10.1136/adc.86.1.44 PMC1719043

[38] Hughes AE, Orr N, Patterson C, Esfandiary H, Hogg R, McConnell V, et al Neovascular age-related macular degeneration risk based on CFH, LOC387715/HTRA1, and smoking. PLoS Med. 2007;4: e355 18162041 10.1371/journal.pmed.0040355 PMC2222948

[39] Wigginton JE, Cutler DJ, Abecasis GR. A note on exact tests of hardy-weinberg equilibrium. Am J Hum Genet. 2005;76: 887–893. 15789306 10.1086/429864 PMC1199378

[40] Purcell S. PLINK. 2009; v1.07.

[41] Purcell S, Neale B, Todd-Brown K, Thomas L, Ferreira MA, Bender D, et al PLINK: A tool set for whole-genome association and population-based linkage analyses. Am J Hum Genet. 2007;81: 559–575. 17701901 10.1086/519795 PMC1950838

[42] Tedesco F. Inherited complement deficiencies and bacterial infections. Vaccine. 2008;26: I3–I8. 19388157 10.1016/j.vaccine.2008.11.010

[43] Costa I, Pajon R, Granoff DM. Human factor H (FH) impairs protective meningococcal anti-FHbp antibody responses and the antibodies enhance FH binding. MBio. 2014;5: e01625–14. 10.1128/mBio.01625-14 25161192 PMC4173785

[44] Prymula R, Esposito S, Zuccotti GV, Xie F, Toneatto D, Kohl I, et al A phase 2 randomised controlled trial of a multicomponent meningococcal serogroup B vaccine (I): Effects of prophylactic paracetamol on immunogenicity and reactogenicity of routine infant vaccines and 4CMenB. Hum Vaccin Immunother. 2014;10: 2005–2014. 10.4161/hv.29218 25424810 PMC4186018

