# Supplementary material for: Susceptibility to Invasive Meningococcal Disease: Polymorphism of Complement System Genes and Neisseria meningitidis Factor H Binding Protein
Source: PLoS One. 2015 Mar 23;10(3):e0120757. doi: 10.1371/journal.pone.0120757 (PMC4370764; doi:10.1371/journal.pone.0120757)
Supplement: S1 Table — (DOCX) [file pone.0120757.s001.docx]

**Table S1. *CFH* PCR and SNaPshot Oligonucleotides.**

| rs419137-F | CAGCTATACCACTGATGTAGAGG |
| --- | --- |
| rs419137-R | CCTACTTACTACTCTCCCATAGG |
| rs1061170-F | TTTATCATTGTTATGGTCCTTAGG |
| rs1061170-R | AGTGTACTTACTGACACGGATGC |
| rs6677604-F | ACCAGAGCAGATACAGCAAAAGG |
| rs6677604-R | AAGCACAATACCTCCACAGTAGC |
| rs2284664-F | GTCATCCATCAAGTGCTACAACC |
| rs2284664-R | CAGTGGAAGTATGTGCCCTAAGC |
| rs3753396-F | CACCTCCTGAACTCCTCAATGG |
| rs3753396-R | ACTGGTAAAGTTGTCCACTCTCC |
| rs800292-F | GGATTAAGAGCAACCCATTCTCC |
| rs800292-R | CTGACCAAACATATCCAGAAGGC |
| rs419137-snapF | GACTGACTGACCACCAACCCTGCAGCACATT |
| rs1061170-snapF | CCCTCCTTATTTGGAAAATGGATATAATCAAAAT |
| rs6677604-snapF | AACCCCCCCCCCAGTTGCCCTGAGAAAATGCGAG |
| rs2284664-snapF | CATCATCATCATAGAAAAATACCAGTCTCCATAGATC |
| rs3753396-snapR | AGACAGACAGACAGACATTTGTCCACTCTCCATCAACACA |
| rs800292-snapR | AAAAAAAAAAACCCCCCCCCCCCCCTGGATATAGATCTCTTGGAAAT |
