# Supplementary material for: Susceptibility to Invasive Meningococcal Disease: Polymorphism of Complement System Genes and Neisseria meningitidis Factor H Binding Protein
Source: PLoS One. 2015 Mar 23;10(3):e0120757. doi: 10.1371/journal.pone.0120757 (PMC4370764; doi:10.1371/journal.pone.0120757)
Supplement: S2 Table — (DOCX) [file pone.0120757.s002.docx]

**Table S2. Sequenom iPLEX Oligonucleotides**

| SNP | Primer | Sequence |
| --- | --- | --- |
| rs1013579 | F | ACGTTGGATGACAAAGCCTTCACATCGCAC |
| rs1013579 | R | ACGTTGGATGGTGCAACTTCTCTGACAAGG |
| rs1013579 | UEP | cccaTGTTACCAACAGACCATGC |
| rs1048926 | F | ACGTTGGATGTGTTGGTGCAAATTTTCTGG |
| rs1048926 | R | ACGTTGGATGATTAGTCTTGGCAGCAGAAG |
| rs1048926 | UEP | TGGCAGCAGAAGGAACAG |
| rs1063499 | F | ACGTTGGATGTTTCCAGCAGGATTGTCCAG |
| rs1063499 | R | ACGTTGGATGAGGAGAACCGTTCATCAGAG |
| rs1063499 | UEP | cccGCTTCATATCTGGCCTTA |
| rs11118516 | F | ACGTTGGATGCATGGATGAATGGAGTTGGC |
| rs11118516 | R | ACGTTGGATGGCATGGATCTCTCAGGTAAC |
| rs11118516 | UEP | ATCTCTCAGGTAACTATCACATG |
| rs1126618 | F | ACGTTGGATGTACTTTCACAGGTAGGAGGG |
| rs1126618 | R | ACGTTGGATGCATGCCTCCATTTCTTGCAC |
| rs1126618 | UEP | GTGGAGAATGAAACAATAGG |
| rs12067507 | F | ACGTTGGATGCCTTCACATCGCACTTGACT |
| rs12067507 | R | ACGTTGGATGAACCGTGCAACTTCTCTGAC |
| rs12067507 | UEP | aTCTCTGACAAGGAAGTC |
| rs12085435 | F | ACGTTGGATGTCACTTTGACTACTGATGCC |
| rs12085435 | R | ACGTTGGATGAAAATGGCAAGCAAGTCTGG |
| rs12085435 | UEP | TGGTTTCAGTTTTGGTTTTAAAATAC |
| rs12568382 | F | ACGTTGGATGGTATTCCACTAAGAGTCTCC |
| rs12568382 | R | ACGTTGGATGGGTTTTATAAAGATGTCTATTG |
| rs12568382 | UEP | tgCTATTGTTATACTAAAAGTGTGAC |
| rs1342440 | F | ACGTTGGATGCATTCTGAGGTGCTGGATTG |
| rs1342440 | R | ACGTTGGATGTGGAGCTCCTGGTCTGTATG |
| rs1342440 | UEP | TCCAGGAAAGGAGAAGA |
| rs17020993 | F | ACGTTGGATGGGCCACTAAAAAATGCATGTC |
| rs17020993 | R | ACGTTGGATGGATGTTTTCCAATAGCAGGG |
| rs17020993 | UEP | cctcCCAATAGCAGGGGTATACAATC |
| rs17216529 | F | ACGTTGGATGGCAGAGTTTTTGATTATATC |
| rs17216529 | R | ACGTTGGATGCTGGCTTCAAGTCGTCATTC |
| rs17216529 | UEP | ttacGTCGTCATTCAACGAATAAA |
| rs17612 | F | ACGTTGGATGGAAGCATTCACAACACGATT |
| rs17612 | R | ACGTTGGATGTTGCCTACTGGAATCAGTGC |
| rs17612 | UEP | gaattGAATCAGTGCAAATGAAGA |
| rs1800963 | F | ACGTTGGATGAGAAACACTGGGAGCTGTGG |
| rs1800963 | R | ACGTTGGATGAGATTCACCTCCAATTCCCC |
| rs1800963 | UEP | gagaaCTGTGATCCCACCCTGCCCCTC |
| rs1801033 | F | ACGTTGGATGGTTAGATCTGTCTTGCGTCC |
| rs1801033 | R | ACGTTGGATGGGAATGCATGGTTGAAAGGC |
| rs1801033 | UEP | ggGAAAGGCTACCAGAGGC |
| rs2071006 | F | ACGTTGGATGATCAGCACCATCCAGCCCAA |
| rs2071006 | R | ACGTTGGATGTACCCCCCCCAACTTCTAC |
| rs2071006 | UEP | CCCCAACTTCTACCTGCTG |
| rs2298711 | F | ACGTTGGATGTGTATCAAGAGACTTTCTG |
| rs2298711 | R | ACGTTGGATGTTCCACTGACCAGTCTGACG |
| rs2298711 | UEP | CAGTCTGACGGTTTCACTCA |
| rs2796267 | F | ACGTTGGATGAAAACAAGACGGCCCCGAGA |
| rs2796267 | R | ACGTTGGATGGTCAGGCATTATTGGGAAC |
| rs2796267 | UEP | TGAGTTTTTCTTGCTTGAATG |
| rs2796268 | F | ACGTTGGATGTTGCTAAGCCCTTGGCCTAC |
| rs2796268 | R | ACGTTGGATGGTCACAAATATGACGGCGAG |
| rs2796268 | UEP | gatgcCGGCGAGCCAGTCCTTTCCC |
| rs2796270 | F | ACGTTGGATGCCAGTCACATGCGCTTGGA |
| rs2796270 | R | ACGTTGGATGGGCTCCAGCACCAATTTAAC |
| rs2796270 | UEP | TTTCCAATCCAGCAGCACA |
| rs2796278 | F | ACGTTGGATGATGAATGAAGGCTTTTTCCC |
| rs2796278 | R | ACGTTGGATGCAGTCCTATGTCAGATAGAAG |
| rs2796278 | UEP | gggATGTCAGATAGAAGTGGCAAAC |
| rs2808467 | F | ACGTTGGATGCCAGGTTTCATTCAAGAGGC |
| rs2808467 | R | ACGTTGGATGCACATCTGGCTTACGGAAAC |
| rs2808467 | UEP | TTACGGAAACAGGTTTCCAAA |
| rs2842707 | F | ACGTTGGATGGTACCTTCCATTAGAATTTGC |
| rs2842707 | R | ACGTTGGATGGAGGTAAGTGAGTGAAGAG |
| rs2842707 | UEP | AGTGAAGAGAAAGAAATACTCAG |
| rs2842754 | F | ACGTTGGATGAAAGGCTACTGCTCACCTTG |
| rs2842754 | R | ACGTTGGATGGAAGTAGCAACCTGCCTTAG |
| rs2842754 | UEP | ACCTGCCTTAGTAGGTAA |
| rs35949016 | F | ACGTTGGATGGGAGACTGTATTTAGGCCTG |
| rs35949016 | R | ACGTTGGATGTCCTTACCTGACCAGTGTCC |
| rs35949016 | UEP | tttgGGAGATGCCAGATCTGTCT |
| rs3737392 | F | ACGTTGGATGTCCTCTGTTAAGGAAGAGAC |
| rs3737392 | R | ACGTTGGATGGAAATCAAGCAGCTGAACCG |
| rs3737392 | UEP | CCGGCAGTTAGATATGA |
| rs3748671 | F | ACGTTGGATGCAAGACTCCCCAAAATGGGT |
| rs3748671 | R | ACGTTGGATGAGAGGCACATGCATCAAGTC |
| rs3748671 | UEP | gggGTAGCTACAAAAGGGAACTTA |
| rs3760776 | F | ACGTTGGATGGGGAGTGAGAGAGTTTAAGG |
| rs3760776 | R | ACGTTGGATGGCAAACTTCCTGGAATGGAG |
| rs3760776 | UEP | gggttATCTGGAATACTGGTTTTGAG |
| rs3805221 | F | ACGTTGGATGTCAGTTACTCAGGATGTACC |
| rs3805221 | R | ACGTTGGATGTCAGATGCCTCTATCCAAAC |
| rs3805221 | UEP | taCTCTATCCAAACAGAACAGGA |
| rs4142863 | F | ACGTTGGATGGCCCATCACCAAGGTTTATG |
| rs4142863 | R | ACGTTGGATGTCTCAAATGAGTTCAAGAG |
| rs4142863 | UEP | AATGAATGAAGGTTGGATG |
| rs4425986 | F | ACGTTGGATGGCAACTCTAATTTTTGGAAC |
| rs4425986 | R | ACGTTGGATGCCCAGTCAGTTTGGAAGTCT |
| rs4425986 | UEP | ggGGAAGAATGCTTCTACTGGGGGAC |
| rs4571969 | F | ACGTTGGATGTCCTGATCTGCCTCTAGAAG |
| rs4571969 | R | ACGTTGGATGGTCTCCTTTCTATCCTTACG |
| rs4571969 | UEP | gTCCTTACGAAGTGAGAAT |
| rs4844568 | F | ACGTTGGATGGTCCAGAAATAGACCTAGAT |
| rs4844568 | R | ACGTTGGATGGCCTTTACTGATTTAAGATGC |
| rs4844568 | UEP | ACTGATTTAAGATGCTCTTTGATA |
| rs4844573 | F | ACGTTGGATGGTAATAGCGTCCTTTTGTTC |
| rs4844573 | R | ACGTTGGATGGAAGCATGTGGAATGTCTGG |
| rs4844573 | UEP | ggagGTGGAATGTCTGGTAAATTA |
| rs4957361 | F | ACGTTGGATGCAAGCCATTCTGTTTCTAAC |
| rs4957361 | R | ACGTTGGATGCCAGTCCACTGAGATTTTTC |
| rs4957361 | UEP | ggCCTTGATGGACTAGTTAAAG |
| rs626457 | F | ACGTTGGATGGAGCTCCACTACACTTTACC |
| rs626457 | R | ACGTTGGATGGGTTTCATAGCCACTGTTCC |
| rs626457 | UEP | GGTCAAATAACTTTCCTCTCTC |
| rs652785 | F | ACGTTGGATGAGCTGCTCCAGTTCTACAAC |
| rs652785 | R | ACGTTGGATGACTCACCTGTCTCCTTACAC |
| rs652785 | UEP | CTGTCCACACTGTGCTT |
| rs6688299 | F | ACGTTGGATGGCAAGCTAGTTAGGATGACC |
| rs6688299 | R | ACGTTGGATGTTACTCTGAGGTGCGAGTTC |
| rs6688299 | UEP | actAGGTGCGAGTTCACCATCTTTA |
| rs700233 | F | ACGTTGGATGCCAGCAAGAGCAGCATGTCA |
| rs700233 | R | ACGTTGGATGGGATGCTTATTTCTAAAATGC |
| rs700233 | UEP | TGCAACTGCAAAGCTCC |
| rs7144 | F | ACGTTGGATGAGTGGGTTACTCAACCTAAG |
| rs7144 | R | ACGTTGGATGATGGTGCGAAGTGAACACTG |
| rs7144 | UEP | ccccTGAACACTGTAGTCTTGT |
| rs7159888 | F | ACGTTGGATGCAGCAAGGCTGAATTACCTC |
| rs7159888 | R | ACGTTGGATGTTACCGGGCTTATCAGGAAG |
| rs7159888 | UEP | taTATCCAGTGTTGCTGGGAAG |
| rs75563598 | F | ACGTTGGATGTAATGGTGGATCCTAAGGTG |
| rs75563598 | R | ACGTTGGATGCTGGGAATCCAGTTCATGGC |
| rs75563598 | UEP | ATCCAGTTCATGGCTATTTT |
| rs947636 | F | ACGTTGGATGATGAGCTTGATGCTGCTTGG |
| rs947636 | R | ACGTTGGATGTAGATACCTCAAACTAAAC |
| rs947636 | UEP | tTACCTCAAACTAAACAAGACCAT |
