# Supplementary material for: Susceptibility to Invasive Meningococcal Disease: Polymorphism of Complement System Genes and Neisseria meningitidis Factor H Binding Protein
Source: PLoS One. 2015 Mar 23;10(3):e0120757. doi: 10.1371/journal.pone.0120757 (PMC4370764; doi:10.1371/journal.pone.0120757)
Supplement: S3 Table — (DOCX) [file pone.0120757.s003.docx]

**Table S3. fHBP oligonucleotides**

| Primer Name | Sequence |
| --- | --- |
| C1D1-F | GGCGTTCGGTTCAGACGATG |
| C2D2-F | CCCCAAGCATTCAGCTCCGACGACC |
| C2D1-F | CCCCCCCCCAAGCATTCAGCTCCGACGATG |
| Common-Fam-labelled-R | GWTTTCARRTGTTCGATTYTGCC |
| Deletion-A-R | CGGCTGAYYTTGTCGTTCTTC |
| Deletion-A-Fam-labelled-F | GCRCCGCTCGACCATAAAGAC |
